# Blastocyst formation, embryo transfer and breed comparison in the first reported large scale cloning of camels

**DOI:** 10.1038/s41598-021-92465-9

**Published:** 2021-07-12

**Authors:** P. O. Olsson, A. H. Tinson, N. Al Shamsi, K. S. Kuhad, R. Singh, Y. B. Son, Y. Jeong, Y. W. Jeong, L. Cai, K. Sakaguchi, S. Kim, E. J. Choi, X. Yu, W. S. Hwang

**Affiliations:** 1 UAE Biotech Research Center, 30310 Al Wathba, Abu Dhabi, United Arab Emirates; 2 Presidential Camels and Camel Racing Affairs, P.O. Box 17292, Al-Ain, United Arab Emirates; 3 Laboratory of Theriogenology, Department of Clinical Sciences, Faculty of Veterinary Medicine, Hokkaido University, Sapporo, Hokkaido 060-0818 Japan; 4 Institute of Cell Biology, School of Biological Sciences, College of Science and Engineering, University of Edinburgh, The Hugh Robson Building, 15 George Square, Edinburgh, EH8 9XD UK; 5 Laboratory Animal Center, College of Animal Science, Jilin University, Changchun, China

**Keywords:** Cloning, Biotechnology, Cell biology, Reproductive biology

## Abstract

Cloning, through somatic cell nuclear transfer (SCNT), has the potential for a large expansion of genetically favorable traits in a population in a relatively short term. In the present study we aimed to produce multiple cloned camels from racing, show and dairy exemplars. We compared several parameters including oocyte source, donor cell and breed differences, transfer methods, embryo formation and pregnancy rates and maintenance following SCNT. We successfully achieved 47 pregnancies, 28 births and 19 cloned offspring who are at present healthy and have developed normally. Here we report cloned camels from surgical embryo transfer and correlate blastocyst formation rates with the ability to achieve pregnancies. We found no difference in the parameters affecting production of clones by camel breed, and show clear differences on oocyte source in cloning outcomes. Taken together we demonstrate that large scale cloning of camels is possible and that further improvements can be achieved.

## Introduction

While camels (*Camelus dromedarius*) have been bred for centuries, embryo transfer (ET) in camels was not pioneered until the early 90’s^1^. The first technical camel “clones” were produced in 2001 from a bisected embryo being transferred to two surrogates, creating identical twins^2^. True cloning, via nuclear transfer to donor oocytes, was achieved in Dubai in 2009^3^. While ET has been widely available, especially in recent years with a number of commercial operations throughout the Emirates, cloning success has been sporadic and isolated. Cloning is generally useful as a proliferation technique, which allows for the increased production of specific genetic traits and desired attributes of individuals and populations^4^.

There is renewed interest in camelid cloning, with various techniques being attempted^5–7^. Eleven healthy mature individuals were selected from the three interest groups (breeds), and tissue samples were collected for culture. Five retired racing camels, three dairy and three show (Beauty), camels were selected with all but two of the eleven beauty camels being female. The objective of this manuscript is to report on the methods and results of a large scale camel cloning attempt and the differences between embryo development, transfer methods, camel breeds and the production of cloned offspring.

The world’s first cloned camel, Injaz, Arabic for achievement, was reported born in April of 2009 in a paper published by Wani et al. in 2010. Wani et al*.* later produced a Bactrian camel (*Camelus bactrianus*) clone through interspecies nuclear transfer using a Dromedary camel as both oocyte donor and surrogate^3,8^. The first Bactrian camels produced through interspecies embryo transfer by Niasari-Naslaji et al*.* showed that this was likely possible, as the barrier to interspecies cloning appears to be primarily with surrogate gestation, and not the developmental competence of embryos^9^. Here we investigate potential large scale cloning of three different Dromedary breeds: racing, beauty, and dairy. We additionally explored the difference in potential donor cell breed or other effect on the production of cloned offspring and of oocytes sources from oocyte donors and from ovaries obtained from abattoirs, as previously reported^10^ and comment on additional points for potentially increasing cloning efficiency. This report is the first known report on the large scale cloning of camels and, to our knowledge, report of surgical embryo transfers in old-world camels comparing cloning of the three basic Dromedary breeds.

## Materials and methods

### Chemicals and media

All chemicals were purchased from Sigma (St. Louis, MO, USA), unless otherwise specified.

### Animal care and ethics statement

Procedures were conducted during the local breeding season, between December and April. Female camels with ages between 4 and 7 years were supplied appropriate nutrient, and given water ad libitum. All animal procedures were conducted in accordance with the animal study guidelines after approval of the Management of Scientific Centers and Presidential Camels (MSCPD) (Accession No: PC4.1.5) and with Animal research: reporting of in vivo experiments (ARRIVE) guidelines.

### Selection and preparation of donor and recipient camels

A large group of approximately 250 camels were selected on the basis of a normal breeding history and the absence of abnormalities in the reproductive tract based on an ultrasonic examination^11,12^. To obtain a workable daily number and adequate ET recipient to egg donor ratios, camels were separated into groups of 7 animals, 3 donor and 4 recipient animals each day with a total of 49 for each week. The techniques of batching the groups and synchronising the animals have been previously described in other works^13,14^. Oocyte donor camels received PMSG (Ceva, Libourne, France) in a single 5000 IU intermuscular bolus injection, recipient camels received a 1500 IU in the same manner. Camels also received 500 µg Closprostenol (Jurox, Rutherford, Australia) at the same time as the PMSG. Donor camels were treated with a 7-day declining dose of Follicle stimulating hormone (FSH) (Folltrophin-V, 400 mg NIH, Vetoquinol, Paris, France) as describe by McKinnon et al. (1994). On the 9th day 100 ug of Gonadorelin Acetate I.V. (Vetoquinol, Paris, France) was administered and an ultrasound was performed to detect for a superovulatory response for OPU collection and at 6 days following ovulation for the presence of corpus luteum for recipient selection^11^.

### Transvaginal ultrasound guided ovum pick up (OPU)

Collection techniques for the donors on Day 10 following administration of synchronization were categorized by ultrasonic examination on Day 9. It was preferable for transvaginal OPU that follicles were between 10 and 20 mm in diameter. Oocytes were obtained via follicular aspiration, only ovaries with a minimum of 5 follicles greater than 10 mm in diameter were attempted. Donors were sedated and prepared by using 0.5 ml of both Ketamine (Ilium, Glendenning, Australia) and Xylaxine hydrochloride (Ceva, Libourne, France) given intravenously and with a 5 ml lidnocaine (Ilium Lignocaine 20, Troy Laboratories, Glendenning NSW Australia) epidural injection^15^. After, the oocytes were aspirated by an Aloka ultrasound unit (Aloka, Tokyo, Japan) with 5 MHz convex transvaginal probe mounted with a needle guide (Aloka, Tokyo, Japan) was placed in position behind the camel. The OPU needle was inserted into follicles of 10 to 15 mm size attached via vacuum line to a 50 ml caped tube with 2 ml OPU Solution (IVF Bioscience, Falmouth, UK) using a regulated vacuum pump. Follicular fluid was transferred to 150 mm diameter Petri dishes for oocyte collection under a stereomicroscope.

### Oocyte collection from abattoir ovaries

Abattoir ovaries were collected daily during the experimental duration, from December through April, at the Al-Ain Municipal slaughterhouse and held at 37 °C for following collection and transported to the laboratory in a 0.9% saline solution. Cumulus oocyte complexes (COCs) were recovered from antral follicles 2 to 6 mm in diameter by aspiration with an 18-gauge hypodermic needle attached to a 10 ml disposable syringe. Grade A and B COCs, those with homogenous cytoplasm and enclosed by at least three layers of compact cumulus cells, were selected and washed three times in Dulbecco’s phosphate buffered saline (DPBS; Welgene, Gyeongsan, KR) supplemented with 5 mg/ml bovine serum albumin (Thermo Fisher Scientific, Waltham, MA, USA) and 1% (v/v) antibiotic–antimycotic (Thermo Fisher Scientific, Waltham, MA, USA). For in vitro maturation (IVM), selected COCs were cultured in groups of 20 to 25 per well of 6-well dish for 40 to 42 h in BO-IVM (IVF Bioscience, Falmouth, UK) at 38 °C in 5% CO2 in humidified atmosphere.

### Establishment of donor cells

Each donor cell line, used as nuclear donor cells for SCNT were obtained, by skin biopsy from the ears of 11 healthy camels of unknown age under the owner’s consent. The tissue biopsy was transported to the laboratory at 4 °C in DPBS supplemented with 1% antibiotic–antimycotic. Tissues were washed 2 to 3 times with DPBS and were minced into small (approximately ≤ 1 mm) pieces with a scissors. The minced pieces were digested in Dulbecco’s modified Eagle’s medium (DMEM; Thermo Fisher Scientific, Waltham, MA, USA) containing 0.1% collagenase type IV (Thermo Fisher Scientific, Waltham, MA, USA) at 38 °C in a humidified atmosphere of 5% CO2 for 1 to 2 h. After washing 2 to 3 times in DPBS, cells were cultured in DMEM supplemented with 10% Fetal Bovine Serum (FBS; Thermo Fisher Scientific, Waltham, MA, USA), 1% nonessential amino acids (Thermo Fisher Scientific, Waltham, MA, USA), 0.1% β-mercaptoethanol (Thermo Fisher Scientific, Waltham, MA, USA) and 1% antibiotic–antimycotic at 38 °C, in 5% CO_2_ in humidified air and cultured for 1 to 2 days. Attached cells were maintained in culture for 3–4 days until they approached 90% confluence and then subcultured at intervals of 4 to 6 days. For cell stocks, cells were trypsinized and reconstituted at concentrations of approximately 1 × 10^6^ cells/ml at passage 2 or 3 and were frozen in 1.5 ml cryovials in 70% cell culture medium containing 20% FBS and 10% dimethyl sulfoxide. Cells were cultured and stored for subculture and use as donor cells for somatic cell nuclear transfer (SCNT) as previously described^16–18^.

### Somatic cell nuclear transfer (SCNT)

SCNT was performed according to Kim et al. (2012) with slight modifications^19^. Briefly, cumulus cells of the COCs were removed from oocytes by repeated gentle pipetting in DPBS containing 0.1% (w/v) hyaluronidase. After denuding, MII phase oocytes were stained with 5 μg/ml bisbenzimide for 3 to 4 min before the micromanipulation to detect genetic materials. Stained oocytes were enucleated by aspirating first polar body and MII plate in a small volume (less than 10%) of surrounding cytoplasm using a beveled glass pipette (16 µm, inner diameter) in SCNT working medium supplemented 5 µg/ml cytochalasin B (CB). Somatic cells as nucleus donor were prepared immediately after enucleation, and a single donor cell was microinjected into the perivitelline space of each enucleated oocyte. The donor cell-oocyte couplets were fused in a fusion medium comprising 0.26 M mannitol, 0.1 mM MgSO4, 0.5 mM HEPES, and 0.05% (w/v) BSA with two DC pulses of 1.8 kV/cm for 15 μs using BTX Electro Cell Manipulator 2001 +  (BTX Inc., San Diego, CA, USA). Reconstructed embryos were activated by treatment to 5 μM ionomycin for 3 min and subsequently with 2.0 mM 6-dimethylaminopurine (6-DMAP) in BO-IVC (IVF Bioscience, Falmouth, UK) under a humidified atmosphere of 5% CO_2_ at 39 °C for 4 h. After that, the embryos were cultured in groups of 6 to 8 per droplet oil-covered for 2 days or 7 days before embryo transfer at 38 °C in a humidified atmosphere of 5% CO_2_ and 5% O_2_. Embryo developmental competency to the cleavage and blastocyst stage was evaluated at 2 and 7 days of culture.

### Non-surgical embryo transfer

Recipients were synchronized and embryos transferred at day 6 following ovulation (3. Selection and preparation of donor and recipient camels). Embryos cultured for 7 days following SCNT, which had reached either expanded or hatched blastocyst stages, were moved to transfer media (IVF Bioscience, Falmouth, UK) and held not more than 2 h at 38 °C until transferred into the recipients. Embryos were transferred ipsilateral to the horn of the uterus with the ovary presenting the best corpus luteum^13^.

### Surgical OPU and embryo transfer

Donors were prepared for aseptic flank laparotomy initially being walked into a specially designed padded crush and sat in sternal recumbency. Sedation was achieved with an intravenous injection of both 100 mg of Ketamine and 100 mg of Xylazine. An inverted “L” pattern of 15 cm × 20 cm was infiltrated with local anesthetic on the left flank of the abdomen in front of the anterior crest of the ilium. The surgery site was shaved and prepared for sterile surgery, an electrocautery patient return electrode was stuck to an appropriately shaved area on the dorsal left hind leg with a disposable surgical drape placed over the surgical site. Using sterile gloves and instruments the skin and muscle were cut open using a combination of scalpel blade, surgical scissors and electro cautery (Olympus, Tokyo, Japan) to gain access to the peritoneal cavity and the left ovary. After exteriorization of the left ovary and aspiration the follicles present, using a standard 18-gauge needle with a 10 ml syringe, the fimbriae end of the oviduct was located. On day 2 of culture, 2 to 4-cell-stage embryos were shipped within 2 h in transfer medium at 38 °C to the site of transfer. Two to three embryos were loaded into a catheter (Sherwood Medical, St. Louis, Missouri, USA) with a minimum medium volume (2 to 4 µL) and gently transferred into the distal 1/4 of the oviduct through the infundibulum.

### Pregnancy diagnosis

A male camel was introduced to the group of surrogate recipients to test for behavioral indicators of pregnancy, such as the tail reflex around 10 days post ET and blood samples for progesterone were taken from all animals around 16 days post transfer. Camels with serum progesterone > 1 ng/ml were then submitted to ultrasound for definitive pregnancy confirmation. Camels with serum progesterone < 1 ng/ml were re-tested via ultrasound prior to being reintroduced into the rotation of animal for preparation the following week. Progesterone analysis was done on serum using Chemiluminescence Immunoassay (Roche, Basel, Switzerland).

### DNA confirmation of clones

Cloned calf parentage was confirmed alongside donor cells and surrogates using the standard procedure of short tandem repeat (STR) profiling was carried out using 17 camelid specific microsatellites (Supplemental Table S1). DNA was isolated from tissues (blood, umbilical cord or placenta) of the clones as well as the surrogate mothers using the DNA isolation kit from Qiagen with minor modifications (Qiagen DNeasy Blood and Tissue Kit). Unrelated female Dromedary camel was used as a control. The PCR conditions were as follows: The 17 microsatellites used in the matching test were grouped into four multiplexes of 11and 6 loci. The PCR conditions of the multiplexes included initial denaturation at 94 °C for 5 min followed by denaturation at 94 °C for 1 min, annealing at 55 °C for 1 min and extension at 72 °C for 45 s. Final extension was carried out at 72 °C for 10 min. The fragment analysis was carried out using ABI 3130 XL and alleles were scored using Gene Mapper Ver 4.0.A representative STR matching is given (Supplemental Fig. SF1).

### Statistics

Statistical analysis was performed using SPSS version 23 (IBM) by one-way analysis of variance (AVONA). To evaluate comparison of among the group, Tukey’s test was performed. Unless otherwise noted, all data were represented as mean ± standard deviation and p values less than 0.05 were evaluated statistically significant.

## Results

Follicular aspiration yielded varied amounts of fluid per ovary which ranged from clear to blood tinged with some follicles appearing almost as whole blood. Oocytes were recovered from approximately 50% of transvaginal ultrasound guided OPU and 70% from surgical OPU although specific annotated values were not directly comparable.

OPU resulted in range of follicular fluid per follicle, with a minimum amount of follicular fluid collected of 2 ml (from a left ovary with 7 follicles with diameters of 10-14 mm) to a maximum of 16 ml (left ovary with 8 follicles ranging from 10 to 14 mm in diameter). Fluid varied from clear through to blood tinged and some collection appeared as blood and contained clots. Oocyte recovery per follicle averaged approximately 50% from transvaginal OPU and 70% from surgical OPU although rigid comparison was not possible.

**Figure 1:**
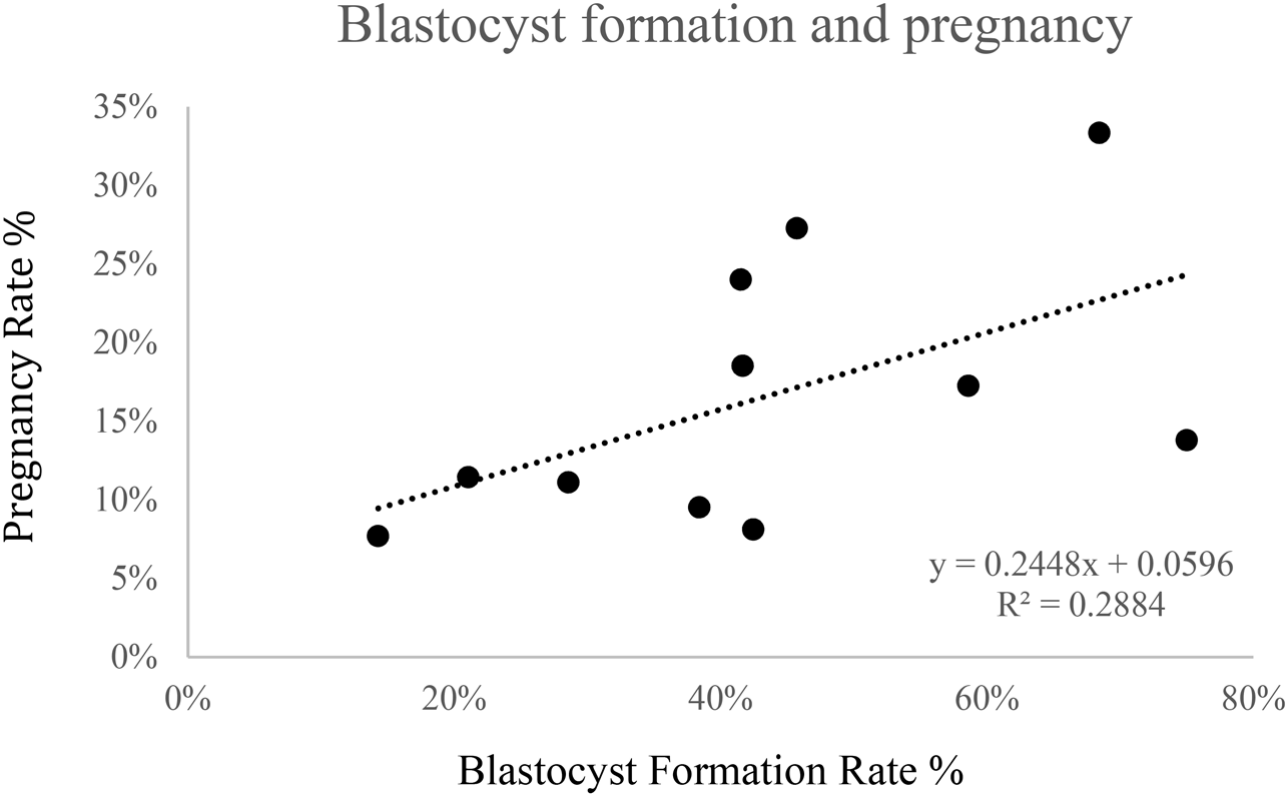
Blastocyst formation rate in OPU derived oocytes versus pregnancy rate from ET of resultant blastocysts, using oocytes obtained from OPU alone.

A comparison of Blastocyst formation rates to Pregnancy rate showed a correlation (R^2^ = 0.288) indicating that blastocysts formation rate could account for a degree of the variation in pregnancy rates (Fig. 1).

Blastocyst formation rate of fused OPU derived oocytes, was compared to pregnancy rates for all 11 individuals (Fig. 1). The cell lines for the nuclear donors showed no statistical difference in overall blastocyst formation rate (Table 1). Blastocyst development did not vary significantly between camel breeds in either oocyte derived from abattoir ovaries, nor did rates differ significantly in blastocyst formation from OPU derived oocytes (Table 1). Rates were however significantly different (P < 0.01) in overall blastocyst rates between the source of the oocytes used, with a greater blastocyst rate, 44.97% ± 28.20 SD, in OPU derived oocytes compared to oocytes obtained from abattoir sources 15.49% ± 22.60 SD. An analysis of oocyte origin on blastocyst development was investigated and shown to differ significantly (Table 1).

**Table 1:** Cellular and oocyte source (OPU and IVM) contribution to fusion, early embryo and blastocyst formation by cell donor and Camel breed.

Cell donor	Dairy camels			Beauty camels			Racing camels				
	M630	M629	M449	B301	B118	B300	R1574	R8257	R1076	R1481	R8633
Oocyte #	228	70	153	181	166	192	181	156	114	53	140
# of replications	12	2	6	7	6	9	6	6	4	3	6
**OPU**											
Fusion rate	63.08%	55.92%	48.48%	57.77%	61.43%	66.03%	67.84%	72.88%	67.77%	58.57%	71.25%
Cleaved oocytes (%)^a^	99 (89.25)	19 (72.02)	64 (79.56)	97 (90.02)	73 (95.10)	75 (62.35)	55 (59.24)	58 (73.56)	35 (42.11)	23 (92.22)	63 (70.08)
Blastocyst rate*^b^	42.45%	14.29%	45.72%	68.44%	41.51%	21.06%	58.60%	28.57%	38.40%	75.00%	41.67%
Ovary/oocyte #	163/671	34/209	48/158	258/1483	75/319	177/812	52/307	56/288	15/63	177/707	38/232
# of replications	14	3	5	11	5	12	6	5	3	7	7
**IVM**											
Fusion rate	57.48%	66.67%	55.80%	50.87%	74.21%	62.90%	54.43%	70.87%	66.67%	66.61%	56.62%
Cleaved oocytes (%)^a^	88 (51.85)	47 (63.27)	9 (26.67)	87 (44.73)	45 (63.60)	87 (51.63)	25 (41.99)	39 (44.19)	10 (52.38)	94 (72.16)	23 (49.98)
Blastocyst rate*^b^	25.27%	17.65%	7.78%	2.74%	35.98%	20.19%	8.13%	n/a	16.67%	25.2%	n/a

Cleaved oocyte percentage calculated from the number of fused oocytes, by replication, including batches where early stage embryos were transferred.

*n/a* early stage embryos were transferred from all batches prohibiting blastocyst rate determination.

*Blastocyst rates calculated from the number of fused oocytes, by replication, excluding batches where early stage embryos were transferred.

^a,b^Denote significant difference (P < 0.005).

The difference between oocytes obtained via IVM and OPU:

- During the experimental period 1191 ovaries were obtained, which yielded 5556 oocytes were subjected to IVM of which 1655 reached maturity (metaphase II) and 1033 (62.42%) were successfully fused to reconstructed oocytes following SCNT. 560 total cleaved embryos resulted from the reconstructed oocytes, 146 embryos were transferred surgically and 102 blastocysts formed. Omitting all data from batches where early stage embryo transfer was performed, blastocysts developed at an average rate of 8.93% from matured oocytes and a rate of 15.49% from fused reconstructed oocytes.
- The total number of oocytes recovered by OPU was 1644 from which 1634 were used to obtain 296 blastocysts at an average rate of 27.02% and 44.97% of fused reconstructed oocytes, batches containing early stage embryo transfer omitted (Table 1).

Over a period of 4 months a total of 287 individual embryo transfers were completed resulting in 47 pregnancies (16.38%). The pregnancy rate did not differ significantly between the groups which varied between 14.03% ± 1.70 SEM for Racing (n = 5), 22.92% ± 6.35SEM for Beauty (n = 3), and 22.92% ± 6.35 SEM for Dairy (n = 3) camels. These pregnancy rates obtained from OPU oocytes alone, to minimize confounding cytoplast variation. Of the resultant 47 pregnancies there were 28 births and 19 calves survived and are presently healthy and thriving (Table 2). Pregnancies were obtained from 85 early stage embryo surgical transfers which resulted in a 12 (14.13%) pregnancies and four births. The remaining 202 transfers were transvaginal blastocyst transfers which resulted in 35 (17.41%) pregnancies and 24 births; a 68.57% pregnancy to birth rate.

**Table 2:** Cellular and breed pregnancy rate and maintenance from cloned ET.

Cell donor	Dairy camels			Beauty camels			Racing camels				
	M630	M629	M449	B301	B118	B300	R1574	R8257	R1076	R1481	R8633
# of surrogates	37	13	22	30	25	35	29	27	21	20	27
Ultrasound pregnancy confirmation	2	1	6	10	6	2	5	3	2	5	5
Resorption (< 3 months)	0	0	3	3	3	0	2	1	2	0	1
Maintained pregnancy (> 5 months)	2	1	3	7	3	2	3	2	0	4	4
Late term loss, stillbirth or calf loss	1	0	2	1	2	1	1	1	0	2	1
Surviving and healthy	1	1	1	6	1	1	2	1	0	2	3

Fusion and Blastocyst Rates from OPU obtained oocytes only.

*Blastocyst Rates calculated from the number of fused oocytes, excluding batches where early stage embryos were transferred.

Comparing embryos derived from OPU oocytes alone, early stage pregnancies obtained from surgical embryo transfer were more than twice as likely to be resorbed than pregnancies obtained from transvaginal blastocyst transfer (Table 2). No difference was observed in either pregnancy maintenance to term or survival comparing surgically transferred embryos from OPU or IVM origin (Supplemental Table S2).

## Discussion

Previous reports on camel cloning have been done using a single or limited number of donor individuals and resulted in few offspring. Here we show 19 healthy clones from 10 distinct donor cell lines. As such this is the first known report addressing the large scale camel cloning. Other camelids have been hybridized with dromedaries but there are no known reports of cloning in the other species, or between camel types other than the Bactrian camel^8^. Several barriers to hybridization have outlined a number of issues related to interspecies reproductive methods, including cloning. Overcoming these interspecies barriers may provide insightful information regarding general ART methods and efficiencies^20^. Here we show a minimum of three individuals from each Dromedary breed, which illustrates that the potential barriers observed in other species, are not likely present between camel breeds.

The economic significance commented on during the initial cloning report by Wani et al. (2010) remains valid today^3^. The developments of camel dairies and the advancements in camel stocks used for racing, dairy and for show continue in many countries, further illustrating this significance. Obtaining quality donor oocytes was the most challenging technical aspect of this project. In our hands IVM oocytes from abattoir samples required 40–42 h to mature, this in contrast to work shown by Wani et al. (2010) and Moulavi et al. (2020), but in line with Wani and Nowshari (2005)^3,5,21^.

The camelid family comprises the Old World camelids (or Dromedary and Bactrian camels) and the New World camelids (llamas, alpacas, guanacos and vicunas). Although the species within each group can hybridize producing fertile offspring, it wasn’t until recently that hybrids have been reported between Old and New World species^9,22^. The capacity for interspecies camelid cloning may assist in conservation of endangered camelids and information gained in one species may be relevant in others as with the Bactrain camel (*Camelus bactrianus*)^8^.

While it is clear that cloning a champion show camel or a high yielding dairy camel may be ideal in terms of long-term “performance”, the situation with racing animals is much more complex, given the many contributing variables required to produce a champion. The first creation of identical twins in camels, from the bisection of embryos, was done to provide for potential research prospects, for racing, in the areas of exercise physiology and nutrition^23^. The creation of multiple clones from a single champion individual could provide a more consistent baseline to investigate the scope of this potential. Furthermore, the potential to compare selected variables in determining non-genetic effectors for racing and dairy camels may additionally become relevant with the ability to routinely produce genetically identical individuals at a relevant scale.

Safe repeatable transvaginal collection was developed as a technique for cattle in hormone stimulated animals in the late 80’s^24,25^. Comparing the results to reports in other animals, including cattle, the recovery rate and total oocytes retrieved were slightly lower in camel transvaginal OPU than in other species^26^. Our recovery rate, which averaged 8.93 ± 6.09 SD oocytes per individual, were however in line with numbers reported in camels^27^. Optimization of protocols and techniques are likely to result in higher recovery rates and increased efficiencies.

Although at present it cannot be ruled out that difference may exist between camel breeds or breeds, individual cell lines can be clearly seen to account for a greater amount of variation than we would expect to find. With this preliminary finding it appears conclusive that if there exist any differences between camel breeds and cloning efficiency it is greatly overshadowed by individual variation (Table 1). Our efficiency data appears to vary from other reports^5,28^ although seasonal variations may play a partial role, they should be considered when making comparisons^29^. The oocyte conditions, both daily and throughout the season may additionally be a factor in our total results. Due to this potential we specifically compare data from OPU derived oocytes to minimize variation in oocyte quality. The comparison of blastocyst rates to pregnancy appears to illustrate cellular contribution to the individual variation observed, potential causal elements are however not yet known. Larger sample sizes would assist in the determination of the factors influencing the variations observed between cells, groups, oocyte source potential and embryo development. The failure to obtain offspring from of one of the 11 cell lines was likely due to the limited pregnancy number compared to reabsorption and loss rates (Table 2). Oocyte and cell conditions are thought to contribute greatly to the success or failure of cloning, and the lack of adequate metrics to determine their potential status, especially in uncommon species, is a continuing challenge. Attempts have been made on a continuing basis to evaluate cell condition as well as oocyte and couplet probability of success after SCNT^5,11,30^. Wani et al. (2018) reported high maturation rates and no difference in pregnancies or delivery between embryos obtained through IVM versus OPU oocytes^10^. Our data indicated both reduced blastocyst potential as well as pregnancy rates from IVM oocytes Surgical transfers ameliorated the initial difference in pregnancy rates, which may provide a solution to the low blastocyst rate from IVM derived oocytes and subsequent challenges, but was made preclusive due to additional animal stress, low potential for replication and lower pregnancy and ultimately birth rates (Supplemental Table S2). From these results it is clear that although surgical methods for oocyte retrieval and early stage embryo transfer may play a role in many species it does not provide an added benefit in the camel.

In addition to oocytes donor cells play critical roles in development of SCNT embryos^10,31^. To better observe the potential donor cell contribution to fusion, blastocyst formation and pregnancy were compared independently of cytoplast source (Table 1). From our findings it is clear that further investigation into the characteristics of both cell and oocytes may yield valuable information regarding cloning capacity and efficiency.

The large late term and delivery to post-delivery loss included 4 stillbirths from cell donors R1481, M449, B300 and R1574; representing all three camel breeds. One M449 calf died two weeks after birth from unknown trauma, a B301 calf died shortly after birth, a R8257 calf died shortly after birth due to dystocia, and another racing camel, R1481, died similarly two days after birth. Necropsies were not performed, primarily due to timing and location of the discovery of the losses. All live born calves were otherwise healthy and all surviving calves have continued to develop normally. The relative size difference of Dairy and Beauty breeds did not appear to have an influence nor were there other factor observed affecting late term loss. The general cause and the influence of cloning on these losses remains unknown. Increased post parturition care is recommended in camel cloning due to the healthy calf losses sustained (Table 2) which may have been averted through increased intervention.

Over the last 30 years there have been a number of stud males that, in terms of the quality of progeny produced, have outshone their rivals. The sudden loss of these genetics due to death or age related illnesses can be devastating to the continued development of these breeds. Overuse of these high demand animals can be detrimental to the bulls concerned. The presence of multiple genetically identical bulls, with proven progeny, has the potential to overcome some of these pre-existing limitations. This may increase commercial breeding potential and provide access to superior genetics and provide increased commercialization. The same or similar techniques may be applied to return diversity lost in endangered camelid species, such as the wild Bactrian camels, as well as species of both commercial and or environmental relevance. Determination of effective methods to increase the efficiency of camelid cloning may further assist in other reproductive technologies and benefit a multitude of species, extending beyond the camelids.

## Conclusion

This was the first large scale camel cloning attempt, which succeeded in the production of healthy calves from 10 distinct donor individuals. The camel breed of donor cells does not appear to differ in the ability to generate blastocysts or to obtain the successful birth of healthy clones. Further work to minimize late term pregnancy loss, and to characterize causal elements is required to optimize the efficiency of the production of cloned camel offspring. Current techniques are adequate to obtain a large number of cloned camels, yet require additional efforts to optimize the process.

## Supplementary Information

Supplementary Legends.

Supplementary Figure S1.

Supplementary Table S1.

Supplementary Table S2.

Supplementary Table S3.
