# Supplementary material for: Blastocyst formation, embryo transfer and breed comparison in the first reported large scale cloning of camels
Source: Sci Rep. 2021 Jul 12;11:14288. doi: 10.1038/s41598-021-92465-9 (PMC8275768; doi:10.1038/s41598-021-92465-9)
Supplement: Supplementary file 1 — Supplementary Legends. [file 41598_2021_92465_MOESM1_ESM.docx]

Supplemental Figure and Table Legend.

Supplemental Figure SF1. Representative STR matching from one cloned camel matched with the cell donor and surrogate as provided by alleles, scored using Gene Mapper Ver 4.0.A by the camel biotechnology center.

Supplemental Table S1. 17 camelid specific microsatellite data including internal ID, locus name, number or repeating units, primers and allele range with original cited reference, as used for cloned parentage confirmations.

Supplemental Table S2. * Co-transfer/embryos with either two different cell donors or oocyte sources. STR matches listed as total matches/all 17 loci. A + 1 and -1 allele variation at R9 and R1 Reference locus was noted for clones 507(B) and 709(0) respectively. Locus reference numbers (Supplemental Table S1). *Italics* designates individuals stillborn or deceased. Surrogate STR matches displayed indicating negative parentage.

Supplemental Table S3. Table as STR data as presented by the Camel Biotechnology Center (CBC) (Supplemental Figure SF1). STR loci are represented with internal Code: RAS#. the SI number (1-17) corresponds to the ID # and locus represented in Supplemental Table S1. STR matches are displayed, for clones and surrogates, in Supplemental Table S2 as exact matches for all 17 loci.
