# Supplementary material for: Blastocyst formation, embryo transfer and breed comparison in the first reported large scale cloning of camels
Source: Sci Rep. 2021 Jul 12;11:14288. doi: 10.1038/s41598-021-92465-9 (PMC8275768; doi:10.1038/s41598-021-92465-9)
Supplement: Supplementary file 2 — Supplementary Figure S1. [file 41598_2021_92465_MOESM2_ESM.pdf]

United Arab Emirates
Presidential Camels and Camel Racing Affairs Centre
Sector of Scientific Centers & Presidential Camels

دولـة الإمـارات العـربيـة المتحـدة
مـركـز شـؤون السـبـاقـات
وهجـن الرئـاسـة
قطاع المراكز العلميـة
وهجـن الرئـاسـة

CAMEL BIOTECHNOLOGY CENTER (CBC)
DNA Fingerprinting Laboratory
Test Report of Clone and Donor Tissue Matching

CC No: 01/2020 **Test Method: STR Matching**

Details of test request	Name	Address	Phone	Email
Contact person	Prof Woo Suk Hwang/Ms Noura Al Shamsi	Project Instructor, Camel Cloning Project/Director Labs	0503815217	hwangws@adbrf.org

Tissue from	Nature of Sample	Sample ID	Lab ID	Sample Received date	Date of Result
Clone	Frozen blood clotted & Umbilical Chord	F412(B)-R8257 F412-R8257	CC No 1	13-4-2020	20-4-2020
Donor	Cultured camel cells P3 (5X10^5)Cells/ ml	R 8257		13-4-2020	20-4-2020
Surrogate	Frozen blood -clotted	S 412 (B)		13-4-2020	20-4-2020

Sl No	STR Loci	Allele Score		
		Donor	Clone	Surrogate
1	RAS 1	165/169	165/169	135/135
2	RAS 2	147/153	147/153	153/174
3	RAS 3	240/240	240/240	238/238
4	RAS 4	261/261	261/261	249/259
5	RAS 5	137/151	137/151	147/153
6	RAS 7	225/225	225/225	223/225
7	RAS 9	144/171	144/171	144/144
8	RAS 10	220/220	220/220	214/216
9	RAS 14	236/240	236/240	236/240

Sl No	STR Loci	Allele Score		
		Donor	Clone	Surrogate
10	RAS 16	107/109	107/109	107/107
11	RAS 24	204/234	204/234	210/234
12	RAS 28	159/159	159/159	159/171
13	RAS 30	285/285	285/285	277/281
14	RAS 41	223/223	223/223	223/239
15	RAS 45	188/224	188/224	192/192
16	RAS 53	260/268	260/268	260/260
17	RAS 54	184/188	184/188	184/188

Final Result: The tissues of Clone F 412 and Donor R8257 perfectly matched at all the loci tested.

The analysis report pertains only to the samples received in the laboratory

Dr T.J. Rasool

Director, Camel Biotechnology Center (CBC)

Dr. T.J.Rasool, Phd
Director
Biotechnology Laboratories
Management of Scientific Centeres
and Presidential Camels

هاتف : ٠٣ - ٧٦٨٦٢٧٨ - فاكس : ٠٣ - ٧٦٨٦٤٦٤ - ص.ب : ١٧٢٩٢ - العيـن - الإمـارات العـربيـة المتحدة
Tel. : 03-7686278 - Fax : 03-7686464 - P.O. Box : 17292 - Al Ain - United Arab Emirates
