# Supplementary material for: Blastocyst formation, embryo transfer and breed comparison in the first reported large scale cloning of camels
Source: Sci Rep. 2021 Jul 12;11:14288. doi: 10.1038/s41598-021-92465-9 (PMC8275768; doi:10.1038/s41598-021-92465-9)
Supplement: Supplementary file 3 — Supplementary Table S1. [file 41598_2021_92465_MOESM3_ESM.docx]

**Supplementary Table S1** Microsatellite loci used for camel clone confirmation

| **ID #** | **Locus** | **Repeat units** | **Primer Sequences** | **Allele range (bp)** | **Reference** |
| --- | --- | --- | --- | --- | --- |
| **1** | **YWLL08** | **Di -Nucleotide** | F: ATCAAGTTTGAGGTGCTTTCC  R: CCATGGCATTGTGTTGAAGAC | **129-175** | **(Lang et al., 1996)** |
| **2** | **VOLP67** | **Di -Nucleotide** | F: TTAGAGGGTCTATCCAGTTTC  R: TGGACCTAAAAGAGTGGAG | **145-208** | **(Obreque et al., 1998)** |
| **3** | **LCA90** | **Di -Nucleotide** | F:TATAACCCTGGTCTCGCCAA R:CCAAGTAGTATTCCATTATGCG | **234-246** | **(Penedo et al., 1998)** |
| **4** | **VOLP10** | **Di -Nucleotide** | F: CTTTCTCCTTTCCTCCCTACT  R: CGTCCACTTCCTTCATTTC | **240-269** | **(Obreque et al., 1998)** |
| **5** | **LCA 33** | **Di -Nucleotide** | F:GAGCACAGGGAAGGATATTCA; R:ACAGCAAAGTGATTCCATAATACA | **135-169** | **(Penedo et al., 1998)** |
| **6** | **LCA18** | **Di -Nucleotide** | F: TCCACCCATTTAGACACAAGC R: TAGGAAGCTCCAAGAAGAAAAGAC | **221-229** | **(Penedo et al., 1998)** |
| **7** | **VOLP03** | **Di -Nucleotide** | F: AGACGGTTGGGAAGGTGGTA  R: CGACAGCAAGGCACAGGA | **144-176** | **(Obreque et al., 1998)** |
| **8** | **LCA63** | **Di -Nucleotide** | F: TTACCCAGTCCTTCGTGGG  R: GGAACCTCGTGGTTATGGAA | **198-232** | **(Penedo et al., 1999)** |
| **9** | **LCA 66** | **Di -Nucleotide** | F: GTGCAGCGTCCAAATAGTCA  R: CCAGCATCGTCCAGTATTCA | **224-242** | **(Penedo et al., 1999)** |
| **10** | **YWLL44** | **Di -Nucleotide** | F: CTCAACAATGCTAGACCTTGG  R: GAGAACACAGGCTGGTGAATA | **86-120** | **(Lang et al., 1996)** |
| **11** | **CVRL1D** | **Di -Nucleotide** | F: GAAGAGGTTGGGGCACTAC  R: CAGGCAGATATCCATTGAA | **188-253** | **(Mariasegaram et al., 2002)** |
| **12** | **CVRL05** | **Di -Nucleotide** | F: CCTTGGACCTCCTTGCTCTG  R: GCCACTGGTCCCTGTCATT | **155-185** | **(Mariasegaram et al., 2002)** |
| **13** | **CVRL07** | **Di -Nucleotide** | F: AATACCCTAGTTGAAGCTCTGTCCT  R: GAGTGCCTTTATAAATATGGGTCTG | **270-300** | **(Mariasegaram et al., 2002)** |
| **14** | **LGU49** | **Di -Nucleotide** | **F:** TCTAGGTCCATCCCTGTTGC R: GTGCTGGAATAGTGCCCAGT | **224-260** | **(Sarno et al., 2000)** |
| **15** | **LGU75** | **Di -Nucleotide** | **F:** GATCAGCTTTGGTGGTTGGT R: CACCTCTTCCCCATGCATAA | **184-230** | **(Sarno et al., 2000)** |
| **16** | **P 149** | **Tetra- Nucleotide** | F: ATCAGGCTCCATTTTTGTGG  R: GTCCATCCTCCAGCACCTAA | **256-284** | **(Munyard et al., 2009)** |
| **17** | **PCTD 17** | **Tetra- Nucleotide** | F: CCCTCTCACCTGTCTACTTG  R: GTATTCTGGCATTGGTTTGT | **172-204** | **(Munyard et al., 2009)** |
|  |  |  |  |  |  |
|  |  |  |  |  |  |

REFERENCES

Lang, K.D., Wang, Y. & Plante, Y. Fifteen polymorphic dinucleotide microsatellites in llamas and alpacas. *Animal Genetics* **27**, 293. (1996)

Obreque, V. et al., Characterization of 10 polymorphic alpaca dinucleotide microsatellites. *Animal Genetics* **29**, 461-462 (1998).

Penedo, M.C., Caetano, A.R. & Cordova, K.I. Microsatellite markers for South American camelids *Animal Genetics* **5**, 411-2 (1998).

Penedo, M.C., Caetano, A.R. & Cordova, K. Eight microsatellites markers for South American camelids. *Animal Genetics* **30**, 161-168 (1999).

Mariasegaram, M. et al., Isolation and characterization of eight microsatellite markers in Camelusdromedarius and cross species amplification in C. bactrianus and Lama pacos. Brief note. *Animal Genetics* **33**, 377-405 (2002).

Sarno, R.J., et al., Development of microsatellite markers in the guanaco, Lama guanicoe: utility for South American camelids. *Molecular Ecology* **9**, 1919-1952 (2000).

Munyard, K., et al., Characterization and multiplex genotyping of alpaca tetranucleotide microsatellite markers. *Small Ruminant Research*. **85**, 153-156 (2009).
