# Supplementary material for: Blastocyst formation, embryo transfer and breed comparison in the first reported large scale cloning of camels
Source: Sci Rep. 2021 Jul 12;11:14288. doi: 10.1038/s41598-021-92465-9 (PMC8275768; doi:10.1038/s41598-021-92465-9)
Supplement: Supplementary file 4 — Supplementary Table S2. [file 41598_2021_92465_MOESM4_ESM.docx]

**Supplementary Table S2** Oocyte source and individual clone confirmation and status

| **Oocyte Source** | **Surrogate** | **Donor Cell line** | **STR matches** | **Surrogate STR matches** | **Clone of:** |
| --- | --- | --- | --- | --- | --- |
| *OPU* | *702(0)* | *R1481* | ***Not tested*** | *not tested* | *R1481* |
| *IVM* | *812(0)* | *R1481* | ***Not tested*** | not tested | *R1481* |
| OPU | 820(0) | R1481 | **17/17** | 1/17 | R1481 |
| OPU | 730(0) | R1481 | **17/17** | 1/17 | R1481 |
| OPU | 727(0) | M449 | **17/17** | 1/17 | M449 |
| *OPU/IVM** | *752(0)* | *M449 / M630** | ***17/17*** | 3/17 | *M449* |
| IVM | 298(B) | M630 | **17/17** | 5/17 | M630 |
| *OPU* | *749(0)* | *B300* | ***17/17*** | 0/17 | *B300* |
| OPU | 540(B) | B300 | **17/17** | 1/17 | B300 |
| IVM | 445(B) | R8257 | **17/17** | 1/17 | R8257 |
| *IVM* | *412(B)* | *R8257* | ***17/17*** | 2/17 | *R8257* |
| OPU | 442(Y) | R8633 | **17/17** | 3/17 | R8633 |
| *OPU* | *269(B)* | *R8633* | ***17/17*** | 5/17 | *R8633* |
| OPU | 838(0) | M629 | **17/17** | 2/17 | M629 |
| OPU | 507(B) | R8633 | **16/17** | not tested | R8633 |
| OPU | 94(Y) | R8633 | **17/17** | 2/17 | R8633 |
| OPU | 804(0) | B118 | **17/17** | 2/17 | B118 |
| OPU | 287(B) | B118 / B301* | **17/17** | 1/17 | B301 |
| OPU | 429(B) | B118 / B301* | **17/17** | 3/17 | B301 |
| OPU | 534(B) | B301 | **17/17** | 1/17 | B301 |
| OPU | 283(B) | B301 | **17/17** | 1/17 | B301 |
| *OPU* | *251(B)* | *B301* | ***17/17*** | 0/17 | *B301* |
| OPU | 253(B) | B301 | **17/17** | 2/17 | B301 |
| OPU | 708(0) | B301 | **16/17** | 4/17 | B301 |
| *OPU* | *388(R)* | *M449* | ***17/17*** | 3/17 | *M449* |
| OPU | 260(B) | R1574 | **17/17** | 0/17 | R1574 |
| OPU | 400(B) | R1574 | **17/17** | 3/17 | R1574 |
| *OPU/IVM** | *736(0)* | *R1574* | ***17/17*** | 2/17 | *R1574* |
|  |  |  |  |  |  |

* Co-transfer/embryos with either two different cell donors or oocyte sources.
 STR matches listed as total matches/all 17 loci. A + 1 and -1 allele variation at R9 and R1 Reference locus was noted for clones 507(B) and 709(0) respectively. Locus reference numbers (Supplemental Table S1). *Italics* designates individuals stillborn or deceased. Surrogate STR matches displayed indicating negative parentage.
