# Supplementary material for: Blastocyst formation, embryo transfer and breed comparison in the first reported large scale cloning of camels
Source: Sci Rep. 2021 Jul 12;11:14288. doi: 10.1038/s41598-021-92465-9 (PMC8275768; doi:10.1038/s41598-021-92465-9)
Supplement: Supplementary file 5 — Supplementary Table S3. [file 41598_2021_92465_MOESM5_ESM.docx]

**Supplemental Table S3** Representative STR data for a single clone and its surrogate.

| **Tissue from** | **Nature of Sample** | **Sample ID** | **Lab ID** | **Date of Result** |
| --- | --- | --- | --- | --- |
| **Clone** | Frozen blood clotted and Umbilical cord | F412(B)-R88257 F412-R88257 | CC No 1 | 20-4-2020 |
| **Donor** | Cultured camel cells P3 (4x10^5^) Cells/ml | R8257 |  | 20-4-2020 |
| **Surrogate** | Frozen blood – clotted | S 412 (B) |  | 20-4-2020 |

| **SI No** | **STR Loci** | **Allele Score** | | |
| --- | --- | --- | --- | --- |
|  |  | **Donor** | **Clone** | **Surrogate** |
| **1** | **RAS 1** | 165/169 | 165/169 | 135/135 |
| **2** | **RAS 2** | 147/153 | 147/153 | 153/174 |
| **3** | **RAS 3** | 240/240 | 240/240 | 238/238 |
| **4** | **RAS 4** | 261/261 | 261/261 | 249/259 |
| **5** | **RAS 5** | 137/151 | 137/151 | 147/153 |
| **6** | **RAS 7** | 225/225 | 225/225 | 223/225 |
| **7** | **RAS 9** | 144/171 | 144/171 | 144/144 |
| **8** | **RAS 10** | 220/220 | 220/220 | 214/216 |
| **9** | **RAS 14** | 236/240 | 236/240 | 236/240 |

| **SI No** | **STR Loci** | **Allele Score** | | |
| --- | --- | --- | --- | --- |
|  |  | **Donor** | **Clone** | **Surrogate** |
| **10** | **RAS 16** | 107/109 | 107/109 | 107/107 |
| **11** | **RAS 24** | 204/234 | 204/234 | 210/234 |
| **12** | **RAS 28** | 159/159 | 159/159 | 159/171 |
| **13** | **RAS 30** | 285/285 | 285/285 | 277/281 |
| **14** | **RAS 41** | 223/223 | 223/223 | 223/239 |
| **15** | **RAS 45** | 188/224 | 188/224 | 192/192 |
| **16** | **RAS 53** | 260/268 | 260/268 | 260/260 |
| **17** | **RAS 54** | 184/188 | 184/188 | 184/188 |

Final Result: The tissue of Clone F 412 and Donor R8257 perfectly matched at all the loci tested.

Figure represents STR data as presented by the Camel Biotechnology Center (CBC). STR loci are represented with internal Code: RAS#, the SI number (1-17) corresponds to the ID and locus represented in Supplemental Table S1. STR matches are displayed, for clones and surrogates, in Supplemental Table S2 as exact matches for all 17 loci.
